# Podocyte-Specific Overexpression of Wild Type or Mutant Trpc6 in Mice Is Sufficient to Cause Glomerular Disease

**DOI:** 10.1371/journal.pone.0012859

**Published:** 2010-09-20

**Authors:** Paola Krall, Cesar P. Canales, Pamela Kairath, Paulina Carmona-Mora, Jessica Molina, J. Daniel Carpio, Phillip Ruiz, Sergio A. Mezzano, Jing Li, Changli Wei, Jochen Reiser, Juan I. Young, Katherina Walz

**Affiliations:** 1 Centro de Estudios Científicos (CECS), Valdivia, Chile; 2 Universidad Austral de Chile, Valdivia, Chile; 3 John P. Hussman Institute for Human Genomics, University of Miami, Miami, Florida, United States of America; 4 Institute of Anatomy, Histology and Pathology, School of Medicine, Universidad Austral, Valdivia, Chile; 5 Nephrology Laboratory, School of Medicine, Universidad Austral, Valdivia, Chile; 6 Department of Pathology, Leonard Miller School of Medicine, University of Miami, Miami, Florida, United States of America; 7 Division of Nephrology and Hypertension, Leonard Miller School of Medicine, University of Miami, Miami, Florida, United States of America; 8 CIN (Centro de Ingeniería de la Innovación CECS), Valdivia, Chile; Inserm, France

## Abstract

Mutations in the TRPC6 calcium channel (Transient receptor potential channel 6) gene have been associated with familiar forms of Focal and Segmental Glomerulosclerosis (FSGS) affecting children and adults. In addition, acquired glomerular diseases are associated with increased expression levels of TRPC6. However, the exact role of TRPC6 in the pathogenesis of FSGS remains to be elucidated. In this work we describe the generation and phenotypic characterization of three different transgenic mouse lines with podocyte-specific overexpression of the wild type or any of two mutant forms of Trpc6 (P111Q and E896K) previously related to FSGS. Consistent with the human phenotype a non-nephrotic range of albuminuria was detectable in almost all transgenic lines. The histological analysis demonstrated that the transgenic mice developed a kidney disease similar to human FSGS. Differences of 2–3 folds in the presence of glomerular lesions were found between the non transgenic and transgenic mice expressing Trpc6 in its wild type or mutant forms specifically in podocytes. Electron microscopy of glomerulus from transgenic mice showed extensive podocyte foot process effacement. We conclude that overexpression of Trpc6 (wild type or mutated) in podocytes is sufficient to cause a kidney disease consistent with FSGS. Our results contribute to reinforce the central role of podocytes in the etiology of FSGS. These mice constitute an important new model in which to study future therapies and outcomes of this complex disease.

## Introduction

Focal and Segmental Glomerulosclereosis (FSGS) is a major cause of end-stage renal disease that is increasing in frequency [1]. Up to a fifth of FSGS affected patients have a high risk for progression to end-stage renal disease [2]. While the clinical presentation of FSGS is often heterogeneous, a characteristic early sign of this glomerular disease constitutes any level of proteinuria and a “focal” pattern of injury, meaning a few but not all of the total sampled glomeruli have “segmental” solidification of the tuft caused by an accumulation of extracellular matrix with obliteration of the capillary lumina (sclerosis) [2]. There are two subgroups in the classification of the disease: Primary FSGS (idiopathic) and Secondary FSGS (genetic, virus infection, drug induced or mediated by adaptive structural–functional responses). However, a working classification system which recognizes five histologic subtypes (collapsing, tip, cellular, perihilar and not otherwise specified (NOS)) can be used in the diagnosis of Primary and Secundary FSGS. Typical findings which confirm the diagnosis of FSGS include collapse, hypercellularity, perhilar hyalinosis, thickened membranes and certainly sclerosis [3].

At ultrastructural levels, normal glomerular function requires that the major components of the glomerular filter (the endothelial cells, glomerular basement membrane and podocytes) be intact and able to provide a filtration barrier. Podocyte foot processes and the glomerular slit diaphragm conform critical elements of the glomerular filter [4]. Recent studies in human as well as in mouse models revealed that podocyte plays a central role in FSGS [5]. Moreover, foot process effacement, which is the stereotypic response of the podocyte to injury owing to reorganization of the actin cytoskeleton, is usually a consistent finding in some histologic subtypes of FSGS [3].

Human genetic studies have helped to identify genes that are involved in the development of FSGS such as podocyte-specific gene nephrosis 2 homolog, podocin (*NPHS2*) [6], [7], α-actinin-4 (*ACTN*4) gene that encodes for a ubiquitously expressed cytoskeletal protein [8], Transient receptor potential channel 6 (*TRPC*6) gene which encodes for a calcium channel [9], [10] and INF2 gene which encodes a member of the formin family of actin-regulating proteins [11]. Besides, recently the myosin heavy chain isoform 9 (MYH9) gene has also been described as a major-effect risk gene among African Americans [12].

TRPC6 is a member of the TRP family of calcium channels that contains more than 50 members separated in seven subfamilies of channel subunits that serve a variety of cellular functions [13], [14]. TRPC6 leads to the influx of calcium in direct or indirect response to phospholipase C (PLC)-mediated signals [15], [16]. It can also be directly activated by DAG [17], [18]. In kidney, TRPC6 is enriched in the podocyte foot processes and in collecting ducts, it was also found to be associated with nephrin and podocin [10], both central protein components of the slit diaphragm.

Initially, six different families were identified with distinct mutations in TRPC6 gene [9], [10], all showing a dominant mode of inheritance with adult onset of disease (the clinical symptoms appearing between the third and fourth decade of life) and importantly, with a variable penetrance. Most recently, a novel mutation in this gene which causes an early development of FSGS in children has been discovered [19]. In this report it was also shown that all mutations are gain-of function mutation by either transporting more calcium or displaying impaired inactivation. However, the relevance of TRPC6 for podocyte function, as well as the signaling pathways and cellular functions altered by the FSGS-associated TRPC6 mutations remain unknown.

Human and mouse Trpc6 share 93% and 87% of homology at cDNA and aminoacid level respectively. In order to address the question whether TRPC6 dysregulation in podocytes is sufficient to drive development of FSGS, we have generated transgenic mice that overexpress the wild type protein or two mutated forms (P111Q and E896K, both previously described in FSGS patients) of Trpc6 channel under the regulation of the human podocin promoter (pNPHS2), which have been extensively used in other transgenic mice to direct expression exclusively to the podocytes [20]–[25]. Characterization of independent transgenic lines showed a significant increment of albumin/creatinine ratio in urine samples within non nephrotic levels. Moreover, abnormal glomerular morphology was observed by PAS staining of kidney from albuminuric transgenic mice. Finally, electron microscopy of glomeruli from transgenic mice showed extensive podocyte foot process effacement.

Taking all our results together, we conclude that podocyte overexpression of wild type Trpc6, as well as both mutated forms analyzed, is sufficient to cause a kidney disease consistent with FSGS.

## Results

### Trpc6-HA wild type and mutated forms associated with FSGS are stable and presented a correct subcellular localization

**Figure 1:**
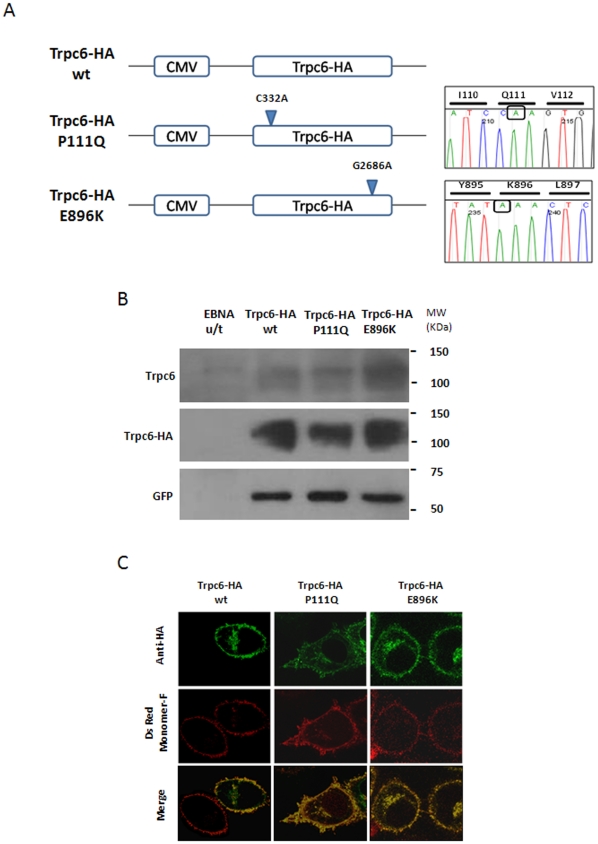
Generation and analysis of modified Trpc6 cDNA's. (A) Schematic representation of the Trpc6 constructs utilized for the in vitro studies. Chromatograms of mutations C332A and G2683A that generate aminoacid substitutions P111Q and E896K are shown. (B) Lysates from EBNA293 either untransfected (u/t) or transfected with a plasmid containing Trpc6-HA wt, Trpc6-HA P111Q or Trpc6-HA E896K cDNA were analyzed by Western Blot analysis with an antibody against Trpc6 (top), against HA (middle) and GFP as a transfection control (bottom). (C) In vitro localization in HeLa cells co-transfected with pDsRed Monomer-F and a plasmid with either Trpc6-HA wt, Trpc6-HA P111Q or Trpc6-HA E896K cDNA were subjected to immunofluoresce to identify HA (green), and processed for direct fluorescence from the farnesylated protein DsRed Monomer-F (red). A representative merged picture shows co-localization of both signals at the plasma membrane for all the transfected proteins. Images were obtained from a confocal microscope (630x).

As a first step towards the generation of Trpc6 transgenic mice we introduced the influenza virus hemaglutinin epitope (HA tag) in frame into the 3′ end of the murine *Trpc6* cDNA clone [26] to be able to distinguish the transgene product from the endogenous protein. The resulting tagged protein was tested for stability, subcellular localization and functionality. In order to determine the generation of the tagged protein product EBNA293 cells were transiently transfected with the *Trpc6*-HA cDNA under the control of the CMV promoter (figure 1A) and 12 h post transfection the cell lysate was run in a 10% SDS-PAGE gel followed by Western blot analysis. As can be seen in figure 1B, a band of the expected molecular weight (∼106 KDa) was observed with an anti-HA antibody in transfected but not in untransfected cell extracts. Further an antibody against Trpc6 revealed a band in the identical position that was also observed in untransfected cell extracts. This result indicates that the HA tag is in frame, that the resulting protein product is stable and of the expected molecular weight. The subcellular localization of the modified protein was assayed by co-transfecting Trpc6-HA and the plasma membrane marker pDsRed Monomer-F into HeLa cells. Trpc6-HA co-localizes with the membrane protein, indicative of correct subcellular localization (figure 1C). We also tested that the addition of the small HA tag did not affect the functionality of the Trpc6 channel. Patch-clamp studies in HEK cells [27] transfected with Trpc6-HA and activated with OAG showed normal activity as a classical inward rectifying channel (data not shown) suggesting that the tagged channel is active.

In order to study two Trpc6 mutant forms previously associated with FSGS in humans we introduced independently the two mutations P111Q and E896K by PCR-based mutagenesis into the Trpc6-HA sequence, as described in materials and methods (figure 1A). These two mutations have been previously shown to have a gain of function when compared to the wild type channel [9], [10].

The stability and subcellular localization of the Trpc6-HA P111Q and Trpc6-HA E896K were validated *in vitro* by transfecting EBNA293 cells with the respective cDNA under the control of the CMV promoter. As can be seen in figure 1B, a band of the same molecular weight of Trpc6-HA wild type was observed with anti-HA and anti-Trpc6 antibodies for both mutant forms, indicating that these mutations do not affect the stability nor the expected molecular weight of the proteins. The subcellular localization of the mutated proteins was also assayed by co-transfecting Trpc6-HA P111Q and E896K independently and each of them with the plasma membrane marker pDsRed Monomer-F into HeLa cells. For both mutant forms the anti-HA signal co-localizes with the membrane marker, indicative of correct subcellular localization for the mutant forms of Trpc6 (figure 1C).

### Generation of podocyte specific Trpc6-HA transgenic mice

**Figure 2:**
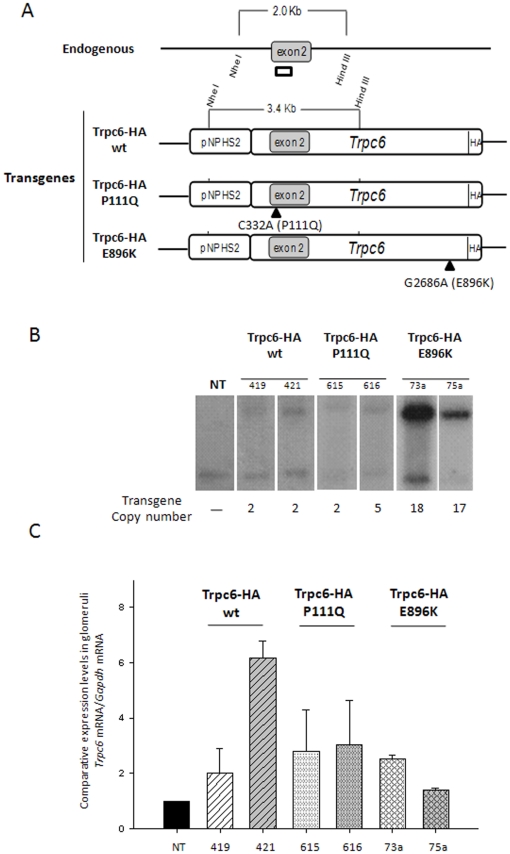
*Trpc6-HA* transgenes and molecular characterization of transgenic lines. (A) Scheme of the microinjected transgenes Trpc6-HA wt, Trpc6-HA P111Q or Trpc6-HA E896K and the wild type allele for *Trpc6*. The complete *Trpc6-HA* cDNAs were subcloned downstream the pNPHS2 podocin promoter, as described in the methods section. The localization of the *Nhe*I and *Hind*III sites surrounding exon 2 on genomic DNA (Endogenous) or the transgene (Transgenes) are depicted. Two distinguishable fragments of 2 or 3.4 kb for the endogenous allele or the transgene, respectively, could be detected by Southern blot analysis with a probe that hybridizes in exon 2 (open rectangle). (B) Southern blot analysis of genomic DNA from non transgenic (NT) or transgenic mice (Trpc6-HA wt, Trpc6-HA P111Q or Trpc6-HA E896K) showed the expected pattern with the designed probe. Transgene copy number for each line was estimated by densitometric analysis with the endogenous *Trpc6* gene for normalization. (C) The glomeruli average expression levels of the transgene by real-time PCR are depicted for each line. Values represent mean +/− SEM.

**Figure 3:**
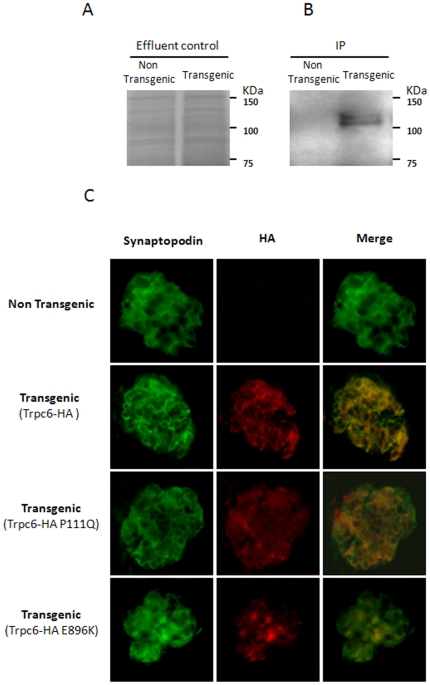
Protein expression in transgenic mice. (A) The *in vivo* Trpc6-HA expression was confirmed by Immunoprecipitation (IP). The IP was performed from isolated mouse glomeruli (n = 8 kidney from each genotype) as described in material and methods. The glomeruli fraction was loaded into an HA affinity column, and a 30 µl aliquot of the sample which did not bind into the column was loaded in a gel and stained with Coomassie brilliant blue as a loading control (effluent control) for transgenic and non transgenic samples. (B) 30 µl of each IP sample was run in a SDS PAGE and a Western blot analysis against HA epitope was performed. (C) Double immunofluorescence of kidney cryosections to detect synaptopodin (green) and HA (red) for every transgenic line utilized in this study (400x).

In order to obtain a podocyte specific transgene, the cDNAs of Trpc6*-*HA wild type, Trpc6*-HA* P111Q and Trpc6*-*HA E896K, were subcloned independently downstream of the pNPHS2 podocin specific promoter [20] (figure 2A). By pronuclear microinjection a total of 17, 5 and 6 transgenic mice were obtained for Trpc6-HA wild type, Trpc6-HA P111Q and Trpc6-HA E896K, respectively. Two founders were randomly selected for each transgene and crossed with C57BL/6J wild type mice: for Trpc6-HA wild type founders 419 and 421, for Trpc6-HA P111Q founders 615 and 616, and for Trpc6-HA E896K founders 73a and 75a. The transgene copy number was estimated by Southern Blot analysis and ranged between 2 and 18 copies in the different lines (figure 2B). The level of expression of each transgene was estimated by quantitative real-time PCR of Trpc6 from mRNA derived of a glomeruli-enriched fraction obtained by sieving technique [28]. Our results demonstrated that Trpc6 in the transgenic lines have a 1.4–6 fold of expression when compared to wild type littermates (figure 2C) (F419 = 2.0+/−0.9; F421 = 6.2+/−0.6; F615 = 2.8+/−1.5; F616 = 3.0+/−1.6; F73a = 2.5+/−0.1; F75a = 1.4+/−0.1). In order to confirm the translation of transgenic mRNA the transgenic protein product was detected by immunoprecipitation (IP) followed by Western blot analysis. Proteins obtained from a glomeruli enriched fraction (a pool of 8 kidneys for each genotype), were immunoprecipitated with an HA-antibody as described in materials and methods and run in a 10% SDS PAGE followed by Western blot analysis. As can be seen in figure 3A–B, only in the fraction derived from transgenic animals we detected a specific band against HA with the expected molecular weight suggesting the correct expression of the transgenic protein. To further confirm this result and to determine the podocyte specific expression of the transgenic proteins we performed co-immunofluorescence of kidney cryostat sections with an antibody directed against a podocyte-specific marker: synaptopodin (Synpo) [28] and the anti-HA antibody to detect the transgenic proteins. As can be seen in figure 3C expression of the transgene was restricted to glomerular podocytes, as illustrated by a strong colocalization with the podocyte-specific marker synaptopodin in all the transgenic lines.

### Albuminuria in transgenic mice

**Figure 4:**
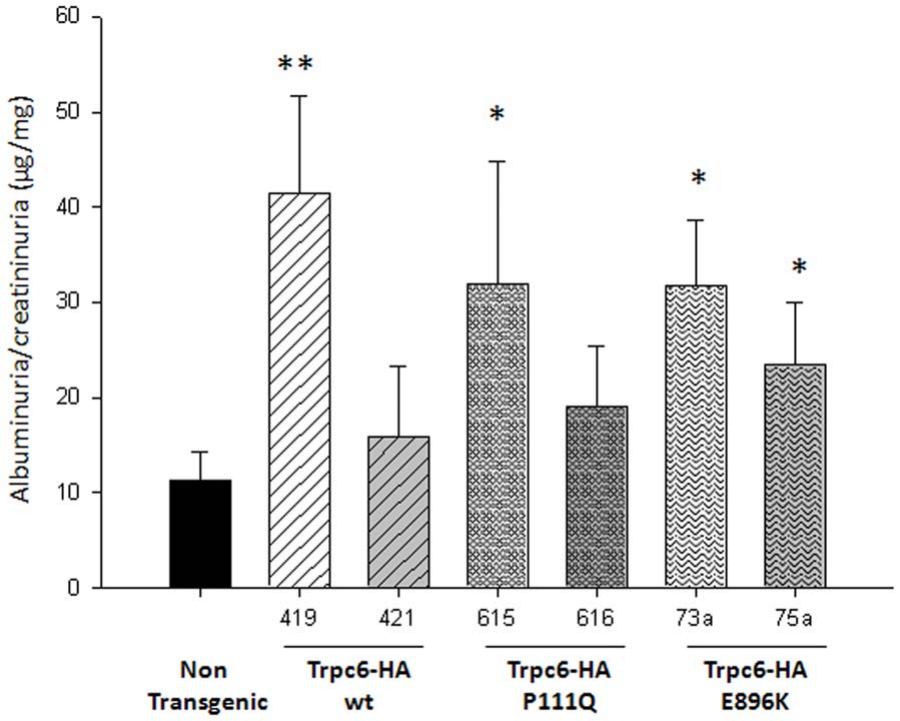
Phenotypic characterization of kidney function in adult transgenic mice. Albuminuria levels (µg/dL) normalized by creatininuria levels (mg/dL) were tested in male mice at the age of 5–9 months. The number of mice analyzed for each genotype is as follows: non transgenic mice: n = 10, transgenic 419 n = 9, 421 n = 9, 615 n = 3, 616 n = 13, 73a n = 17, 75a n = 9. Data are presented in the bars as mean +/− SEM. * and ** mean statistical significance using non transgenic mice as control group with a *P* value <0.05 and <0.01, respectively.

Albuminuria/creatininuria levels were used as an initial screening parameter for glomerular disease in this study. Adult mice (5–9 month of age) were tested for albuminuria/creatininuria in instant fresh urine samples. Characterization of the independent transgenic lines showed an increment of albumin/creatinine ratio in urine samples within non-nephrotic levels. Several transgenic lines appeared significantly albuminuric when compared to the non transgenic littermates (non transgenic: 11.3+/−3.0; 419: 41.4+/−10.3, *P* = 0.004; 615: 32.0+/−12.8, *P* = 0.015; 73a: 31.8+/−6.8, *P* = 0.017; 75a: 23.4+/−6.6, *P* = 0.05) (figure 4). To address the penetrance of this phenotype individual mouse were analyzed and considered proteinuric when the urine albumin/creatinine levels were higher than the average value of wild type littermates plus 2 standard deviations. The percentage of albuminuric mice was different in the individual transgenic lines ranging from 23–45%.

### Structural injuries induced by the expression of Trpc6-HA wild type or mutants P111Q and E896K in podocytes

**Figure 5:**
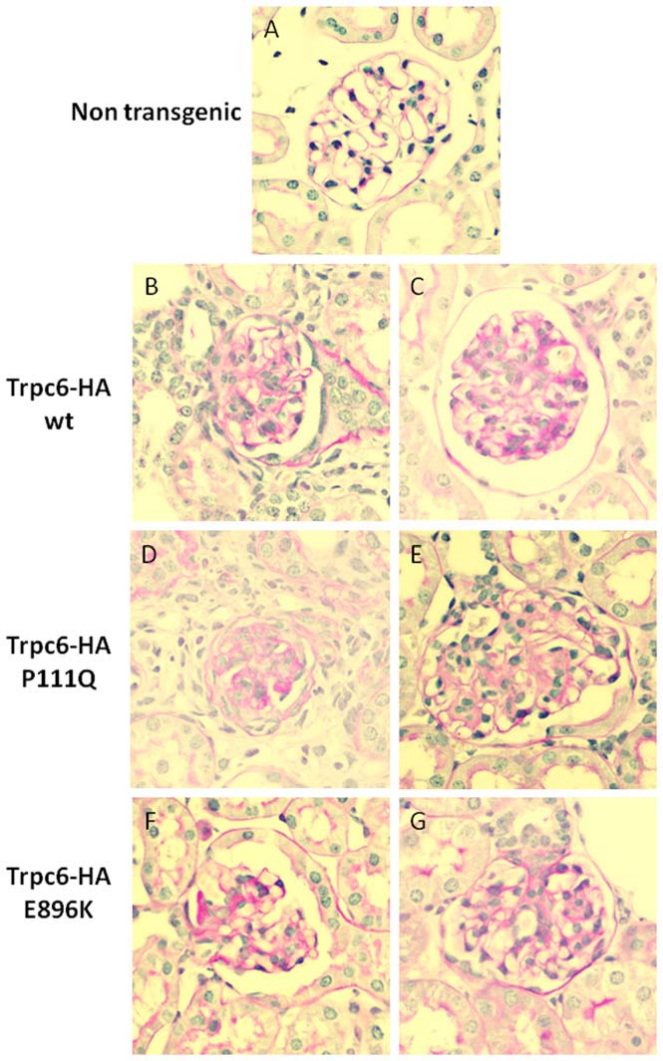
Histopathological lesions analyzed in adult transgenic mice. Representative examples of kidney sections stained with PAS in 400x for all the samples are shown. (A) Non transgenic mouse normal glomerulus, (B) Trpc6-HA wt line 419, (C) Trpc6-HA wt line 421, (D) Trpc6-HA P111Q line 615, (E) Trpc6-HA P111Q line 616, (F) Trpc6-HA E896K line 73a and (G) Trpc6-HA E896K line 75a. The glomerular lesions were analyzed in blind by a pathologist and are summarized in Table 1.

To determine whether the increased albuminuria in transgenic mice was associated with enhanced renal injury, kidneys were systematically examined for the presence of pathological changes in the glomerular, vascular, and interstitial compartments in adult male mice (5–9 month of age). Glomerular lesions in the wild type group were relatively mild (Table 1). In contrast, kidneys from transgenic mice showed more severe pathological findings in glomeruli including hypercellularity, mesangial expansion, and glomerulosclerosis (figure 5 and Table 1). Overall, all transgenic mice lines exhibited perihilar hyalinosis, glomerular sclerosis, glomerular collapse and thickened membrane that are well known pathological features described in different stages of FSGS. As shown in Table 1 and figure 5, the severity of renal pathological abnormalities was significantly increased in transgenic mice compared to control animals (in all cases *P*<0.001). On average, 10–30% of the transgenic glomeruli analyzed had lesions. Tubulointerstitial damage was also observed in transgenic mice and consisted mainly in tubular atrophy, presence of casts, fibrosis as well as inflammatory infiltrates. In conclusion, renal injury was found in all the analyzed lines which support the concept that podocyte-specific expression of wild type or mutant Trpc6 channel is sufficient to drive FSGS-like progressive lesions that extend beyond the level of glomeruli into the tubulo-interstitium.

**Table 1:** Histopathologic injury scores from kidneys of non transgenic and transgenic mice at 5–9 months of age.

		GLOMERULI			TOTAL SCORE
		HC	SCL	MEM	
**Non transgenic**		1.8+/−0.3	0+/−0	0+/−0	1.7+/−0.3
**Trpc6-HA**	**419**	3.0+/−0.3*	2.3+/−0.6**	0+/−0	5.3+/−0.8**
**wt**	**421**	2.7+/−0.4	2.7+/−0.8**	0+/−0	5.4+/−1.2**
**Trpc6-HA**	**615**	3.2+/−0.3**	3.7+/−0.6***	0+/−0	6.9+/−0.9***
**P111Q**	**616**	2.8+/−0.3*	2.8+/−0.6***	0+/−0	5.6+/−0.8**
**Trpc6-HA**	**73a**	2.6+/−0.3	3.4+/−0.6***	0.2+/−0.2	6.3+/−0.9***
**E896K**	**75a**	4.0+/−0***	3.9+/−0.4***	0.6+/−0.3	8.4+/−0.6***

Features consistent with a glomerulopathy found in Trpc6-HA wild type (419 and 421) and mutants P111Q (615, 616) and E896K (73a, 75a) transgenic mice are shown. HC: hypercellularity, SCL: sclerosis, MEM: thickened membranes. The total scores for all glomerular lesions found in each transgenic line is shown in the last column. For each genotype the number of adult mice analyzed is as follows: non transgenic mice: n = 9, transgenic 419 n = 10, 421 n = 7, 615 n = 11, 616 n = 12, 73a n = 11, 75a n = 7. Data are presented as mean +/− SEM.

*; **; *** mean statistical significance using non transgenic as control group with a *P* value <0.05, <0.01 and <0.001, respectively.

**Figure 6:**
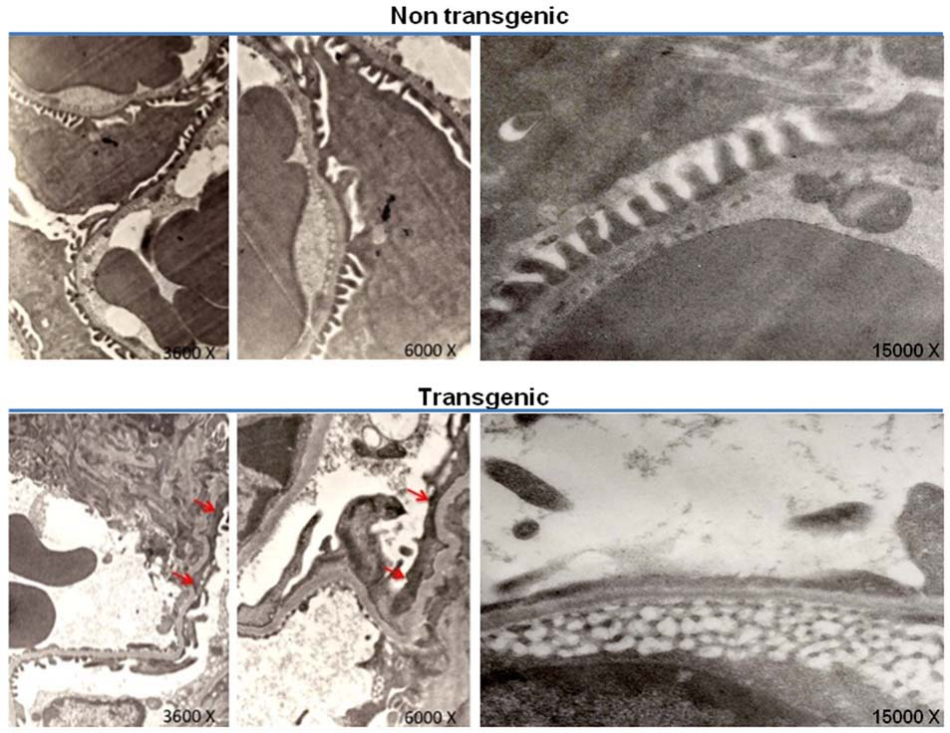
Ultrastructural changes in podocytes from Trpc6-HA transgenic mice. Kidney sections from 3 month old non transgenic (top panel) and transgenic (bottom panel) mice were assessed by electron microscopy. In the top panel an overview of normal mesangium and capillary loops and filtration barrier with normal foot processes is shown for the non transgenic mice (3000, 6000 and 15,000x respectively). In the bottom panel it can be observed that extensive foot process effacement of the podocytes are present in the transgenic section (red arrows) (3000, 6000 and 15,000x respectively).

As ultrastructural changes in podocytes occur at an early stage in several glomerulopathies, we also performed electron microscopy of the transgenic mouse lines at ∼3 months of age. We found normal configuration of podocytes with coordinated interdigitating pattern of foot processes in control littermates. In contrast, the Trpc6 transgenic mice revealed extensive foot process effacement as well as areas with nude glomerular basement membrane where podocyte detachment has occurred (Figure 6). These findings are consistent with progressive glomerular disease such as that seen in human FSGS [29].

## Discussion

FSGS is the second leading cause of renal insufficiency, exceeded only by diabetic nephropathy. In humans, several genes [6]–[12] associated with development of FSGS have been identified, among them the *TRPC6* gene. Distinct mutations in TRPC6 were described in different families all showing a dominant mode of inheritance with adult onset of disease and variable penetrance [9], [10], [19], [30]. Recently, mutations in the TRPC6 channel were found associated with FSGS in non familiar cases, one of them being of pediatric onset [30]. This paper also described that all TRPC6 mutations know to date are gain of function mutations which elevate peak calcium entry or allow the TRPC6 channel to remain open for larger period of time once activated.

In this report we describe the generation of a mouse model of FSGS by overexpressing the wild type or mutant forms of calcium channel Trpc6 exclusively in podocytes. One of our main findings is that the overexpression of the wild type Trpc6 channel is enough to produce a pathologic phenotype. This is interesting because transient overexpression of TRPC6 in the glomerulus leads to an acute onset of proteinuria [31]. In essence, it is irrelevant if mutated or increased wild type TRPC6 is present in podocytes as they both can produce similar phenotypes. This finding raises the question of whether the common downstream phenomenon of dysfunctional TRPC6 is largely a calcium level-mediated effect rather than structural effects of a mutated TRPC6 channel. Future studies need to clarify if overexpression of genes other than Trpc6 that can also increase podocyte calcium entry will cause similar phenotypes.

Another example involving enhanced expression of wild type TRPC6 and disease was found in idiopathic pulmonary arterial hypertension in humans [32].

Consistent with the phenotype observed in patients with kidney disease, our Trpc6 transgenic mice presented albuminuria in adult male mice with variable penetrance observed in the analyzed transgenic lines. Since the different lines all showed comparable incomplete penetrance, it is probably not related to copy number. Furthermore, incomplete proteinuric penetrance was also observed in mice expressing a mutant form of Actn4 [33]. This might be explained by the influence of the environment and/or genetic background since it was already proven that combinations of genetic heterozygosity (bigenic heterozygosity) that alone do not result in clinical kidney disease could function together to enhance susceptibility to glomerular damage and FSGS [34]. Moreover, the phenotype variable penetrance is a well established finding in humans with FSGS related to TRPC6 mutations, suggesting that it may contribute to glomerular diseases in a multi-hit setting [30].

The diagnosis of FSGS is based on the clinical findings of proteinuria and specific histopathological changes, with the scarring (sclerosis) in scattered regions of the kidney of a portion of the glomeruli (focal) and limited to one part of the glomerulus (segmental) [3]. Our results strongly support that familial FSGS associated with TRPC6 mutants are caused by podocyte dysfunction. We observed a number of renal pathologic changes in mice overexpressing Trpc6 in a podocyte-specific manner that are consistent with different stages of FSGS-like phenotype. These include focal perihilar hyalinosis, glomerular collapse, increased mesangial matrix and focal sclerotic glomeruli. This combination of pathologic and clinical data in addition with the early ultrastructural abnormalities found, indicates that mice with podocyte-specific expression of wild type Trpc6 exhibit a phenotype similar to that seen in humans with a gain of function mutation in TRPC6.

Several studies have shown the important role of the podocyte in the etiology of glomerular diseases like FSGS [35], [36]. Mouse knockout models demonstrate that deficiencies of either podocyte-specific genes, encoding for podocin [37], nephrin [38], and Neph1 [39] or more ubiquitously expressed genes, encoding for α-Actn4 [33], CD2AP [40] and Fyn [41] can result in podocyte dysfunction leading to progressive glomerular disease. Transient non-directed overexpression of TRPC6 under the regulation of a CMV promoter leads to an increase of proteinuria in mice, showing a correlation between TRPC6 expressions and filtering abnormalities [31]. However, since Trpc6 is ubiquitously expressed and the reported transient overexpressors do not show any selectivity our mouse models provide novel tools to study the contribution of podocyte dysfunction in the pathogenesis of FSGS. Interestingly, our data shows that podocyte specific Trpc6 manipulations in mice are able to recapitulate the human disease and therefore indicate that podocyte dysfunction is a central element in the development of FSGS. Moreover, this work provides evidence that, in addition to the effects of gain-of-function mutations in the TRPC6 gene, constant elevated levels of wild-type TRPC6 protein is equally powerful to trigger podocyte dysfunction that include not only proteinuria but also FSGS. Supporting this notion, Möller et al. have found a specific increase in the expression of TRPC6 within the glomerulus and podocytes in a variety of glomerular diseases, including minimal change disease, FSGS and membranous nephropathy [31]. Thus, apart from a mutation in the TRPC6 coding and regulatory regions, significant changes in the repertoire of factors that regulate its levels are probably responsible for FSGS and related disorders.

A recent report [42] demonstrates that TRPC6 mutations previously shown to enhance channel activity lead to enhanced basal NFAT-mediated transcription in several cell lines, including cultured podocytes. Activation of NFAT by TRPC6 mutants was blocked by inhibitors of calcineurin, calmodulin-dependent kinase II, and phosphatidylinositol 3-kinase thus identifying the activation of the calcineurin-NFAT pathway as a potential mediator of FSGS. In this scenario, and taking all into consideration, our mouse model can be used to test this hypothesis. On the other hand, the efficiency of antagonists of TRPC channels, i.e. 2APB and SKF96365, can be explored to reduce the kidney damage seen in the Trpc6 transgenic mice.

In summary, we have developed transgenic mice that overexpress the wild type or two mutant forms of Trpc6 calcium channel proteins in a podocyte-specific manner. Our results strongly support the concept that podocyte dysfunction is the principal cause of familial FSGS related with TRPC6 mutations. Moreover, they provide evidence that, in addition to the effects of gain-of-function mutations in TRPC6, elevated levels of wild type Trpc6 protein are sufficient to trigger proteinuria, histological and ultrastructural changes consistent with a FSGS phenotype and podocyte depletion. Furthermore, our mouse model provides an important tool for testing novel therapeutic strategies since channel activities are suitable for manipulations with pharmaceutical drugs that eventually may aid in the prevention and treatment of this disease.

## Methods

### Cloning of murine Trpc6-HA cDNA and generation of Trpc6 mutant forms

The murine *Trpc6* cDNA was kindly donated by Dr. Birnbaumer [26]. In this plasmid we repaired a point mutation A_2615_G that caused an aminoacid substitution D872G and we also simultaneously incorporated the tag hemaglutinin (HA) at the 3′ by site directed mutagenesis with the following primers: Pr1 RepHA *forward* (5′-CAA GTA CAA GGA GCT CAG A-3′); Pr2 RepHA *reverse* (5′-CTT ATC AAT CTG GGC CTG C-3′); Pr3 RepHA *forward* (5′-CAG ATT GAT AAG GAG AGC GA-3′); Pr4 RepHA *reverse* (5′- AAG GAA TTC TTA AGC GTA ATC TGG AAC ATC GTA TGG GTA TCT GCG GCT TTC CTC CAG CT-3′). The PCR product was subcloned in a pGEMT-Easy plasmid and subcloned in the donated pCDNA3 Trpc6 through *Swa* I and *Eco* RI restriction sites, resulting with the pCDNA3 *Trpc6-HA*. To improve the expression of *Trpc6*-*HA* in this pCDNA3 we introduced an alfalfa mosaic virus RNA 4 (AMV RNA4) 5′UTR through *Kpn* I and *Xcm* I restriction sites. The nucleotide substitutions C_332_A (P111Q) and G_2686_A (E896K) were generated independently by site-directed mutagenesis with the following primers: Pr1 C_332_A *forward* (5′- AAC AGA CTG ACT CAC CGG C-3′); Pr2 C_332_A *reverse* (5′-CCA CTT GGA TGT TGC CAT AT-3′); Pr 3 C_332_A *forward* (5′-CAT CCA AGT GGT GCG GAA G-3′); Pr 4 C_332_A *reverse* (5′-AAA GCA TCC CCA ACT CGA GA-3′); Pr1 G_2686_A *forward* (5′-CAA GTA CAA GGA GCT CAG A-3′); Pr 2 G_2686_A *reverse* (5′-GGA GTT TAT AAC GGA GAC TT-3′); Pr 3 G_2686_A *forward* (5′-CCG TTA TAA ACT CCT TGA AG-3′); Pr4 G_2686_A *reverse* (5′-GAG TGT CAT GGA GCT CGA-3′). PCR products were cloned in pGEMT-Easy and subcloned to plasmid pCDNA3 Trpc6-HA through the *Sgr* AI/*Xcm* I (mutant P111Q) and *Swa* I/*Eco* RI (mutant E896K) restriction sites. The mutations were confirmed by restriction analysis with *Bfi* I (mutant P111Q) *and Psi* I (mutant E896K) and by direct sequencing.

### In vitro studies

To determine the ability of pCDNA3 *Trpc6-HA* to express the proteins (wild type or mutant forms) we performed Western blot analysis. EBNA293 cells were transfected with 1.6 µg of the corresponding plasmid using Lipofectamine2000. Sixteen hours post transfection cells were lysed in RIPA buffer containing 1x protease inhibitor cocktail (Sigma). Ten µg of total protein were run in a 10% SDS-PAGE, transferred to a 0.2 µm PVDF membrane, blocked with 5% not fat milk in TTBS (137 mM NaCl, 0.1% Tween 20 and 20 mM TrisHCl, pH 7.6) and incubated with rabbit anti-Trpc6 (ab47679 Abcam), 1/1000. After 3 washes of 30 min each with TTBS the membrane was incubated with HRP-conjugated goat anti rabbit 1/3000 (Pierce) and visualized with enhanced chemiluminiscence. The PVDF membrane was then stripped, blocked and incubated with a rat anti-HA antibody 1/5000 (clone 3F10, Roche) overnight at 4°C. Bound primary antibody was detected with HRP-conjugated goat anti rat 1/40000 (Pierce) and visualized as described above.

To determine subcellular localization of Trpc6-HA proteins (wild type or mutated forms) HeLa cells were co-transfected with pCDNA3 *Trpc6-HA* (wild type, P111Q or E896K respectively) and pDsRed Monomer-F (Clontech) using Lipofectamine 2000 as described above. The cells were fixed 16-20 h postransfection with PFA 4%. Afterwards, the cells were treated with PBS-TX100 0.1% for 15 min and blocked with PBS-gelatin 0.2% for 1 hour. Next, HA tag was detected with a rat anti-HA 1/500 (clone 3 F10, Roche) antibody and with a goat anti rat Alexa Fluor 488 (Invitrogen). Images were captured with a confocal microscope Zeiss LSM710.

Patch-clamp studies in HEK cells transfected with Trpc6-HA and activated with OAG was performed as previously described [27].

### Generation and molecular characterization of transgenic mice

For all the transgene constructs (Trpc6-wild type, Trpc6-HA P111Q and Trpc6-HA E896K) the CMV promoter was replaced with the human podocin promoter (NPHS2) [20] (kindly donated by Dr. Holzman) utilizing the same two step strategy. In the first step a 1.2 kb *Trpc6 Not* I fragment (*Trpc6*-1.2Δ) was subcloned downstream the pNPS2 promoter resulting in the pUC19 NPHS2 *Trpc6-1.2Δ* plasmid. In the second step, the CMV promoter in the pCDNA3 *Trpc6-HA* plasmid was replaced by the pNPHS2 promoter coming from the pUC19 NPHS2 *Trpc6-1.2Δ* plasmid by subcloning it between the *Xba I* and *SgrAI* sites, resulting in the pNPHS2 Trpc6-HA wt, P111Q or E896K plasmids respectively. To confirm the accuracy of the transgenes the plasmids were sequenced before microinjection.

The transgenes containing the NPHS2 promoter, *Trpc6-HA* cDNA (wild type or mutants) and BGH polyadenilation signal were released from the corresponding pNPHS2 *Trpc6-HA* plasmid by digestion with *Bfu*I and purified by gel electrophoresis using the QIAExII Gel Extraction kit (QIAGEN) and Elutips columns (S&S).

Pronuclear microinjection into C57B6/6J × CBA/J zygotes was performed with ∼500 molecules of each transgene. Two founders from each transgene were selected for colony expansion by crossing them with pure C57B6/6J mice to obtain F1 mice. F1 × F1 mating were set up to generate F2 mice that were used for all the phenotypic characterization. Mice were maintained in a SPF facility with a 12 h light:dark cycle (lights on at 7 AM, off at 7 PM) with access to food and water *ad lib*. All testing procedures were approved by the CECS Institutional Animal Care and followed the NIH Guidelines, “Using Animals in Intramural Research”.

#### Genotyping

For all transgenes genomic DNA was isolated from 14–21 day old mice tails and used for genotypic analysis by PCR with: *Trpc6* exon 8 *forward* (5′-GACACTGTTCTGGGCTATCT-3′), *Trpc6* intron 8 *reverse* (5′-CCCATTTTCCTCTCC CCACC-3′) and *Trpc6* 3′UTR *reverse* (5′-CAGTGTGATGGAGCTCGA-3′). The amplification reaction was as follows: 95°C for 5 min (denaturation) followed by 30 cycles of 94°C for 30 sec, 57°C for 45 sec and 72°C for 45 sec, and a final extension step of 72°C for 5 min. The endogenous Trpc6 gene yields a ∼530 bp PCR product while the transgene yields a ∼830 bp PCR product.

#### Southern blot analysis

For all transgenes ten µg of tail DNA were digested overnight with *Nhe*I and *Hind*III, electrophoresed through a 1% agarose gel and transferred to a Hybond C+ membrane by capillarity. A probe that hybridizes to exon 2 of *Trpc6* was generated by PCR with the primers *Trpc6* exon 2 *forward* (5′-AACAGACTGACTCACCGGC-3′) and *Trpc6* exon 2 *reverse* (5′-AAAGCATCCCCAACTCGAGA-3′). The PCR probe was marked with dCTP-αP^32^ using the Megaprime Labeling System (Amersham).

### Determination of transcript expression

We obtained a glomeruli-enriched fraction from 3-month old male mice (n = 4 kidneys from each genotype) utilizing a sieving technique [28]. Total RNA was isolated from these fractions with TRIzol Reagent (Invitrogen) according to manufacturer's instructions. Prior to reverse transcription, RNA samples were treated with rDNAse I (DNA-free kit, Applied Biosystems). Then cDNA was syntethized using ImProm-II Reverse Transcription System (Promega). The possibility of contamination with genomic DNA from the transgene was eliminated by using rDNAseI and ‘no RT’ controls in the reactions of Real Time PCR. We performed quantitative Real Time PCR with QUANTIMIX EASY SYG kit (BIOTOOLS B&M Labs). The primers used for detecting *Trpc6* transcript was designed in exon 2 (*forward* 5′-CATCCCAGTGGTGCGGAAG-3′ and *reverse* 5′-AAAGCATCCCCAACTCGAGA-3′). Results were normalized against *Gapdh* (*forward* 5′- ACCCAGAAGACTGTGGATGG-3′ and *reverse* 5′- CACATTGGGGGTAGGAACAC-3′). All reactions were performed in duplicate with the following amplification parameters: 10 min at 95°C, and 40 cycles of 10 s at 95°C, 30 s at 58°C and 30 s at 72°C. Delta Ct method was used to compare the DCt (cycle threshold) value of transgenic animal samples (Ct of target-Ct of control transcript) with DCt value of wild type mice samples.

### In vivo protein expression analysis

The *in vivo* Trpc6-HA expression was confirmed by Immunoprecipitation (IP) and Immunofluorescence analysis in every line. IP was performed from isolated mouse glomeruli using anti-HA Immunoprecipitation Kit (Sigma, Cat No. IP0010). Isolation of glomeruli was obtained by passing kidney tissue (n = 8 kidney from each genotype) through a series of sieves as described [28]. After IP, a Western blot analysis against HA epitope was performed (primary antibody Rat anti-HA 1∶5000 clone 3F10, Roche). Bound primary antibody was detected with HRP-conjugated goat anti rat 1∶15000 (Santa Cruz Biotechnology, Cat. No. sc-2032).

To confirm that the expression of the transgene was resctricted to podocytes, an immunofluoresce analysis against HA epitope was performed for every line in analysis. Adult mice were perfused with 1x PBS and 4% PFA. Kidneys were dissected and then frozen in OCT medium. Five µm sections were washed three times (5 minutes each) with 1x PBS and incubated during one hour at room temperature with blocking solution (1x PBS, 10% NGS, 0.3% Triton x-100). Additional blocking steps were performed using Super Block and Mouse-to-Mouse Blocking Reagent according to the manufacturer instructions (ScyTek Laboratories). Primaries antibodies (prediluted in blocking solution) were incubated ON at 4°C (Rabbit anti HA 1∶300, Bethyl Cat. No. A190-108A; Mouse anti Synaptopodin 1∶80, Progen Cat. No. 61094). Secondary antibodies were incubated for 40 minutes at RT (Goat anti Rabbit AF568 1∶1000; Goat anti Mouse AF488 1∶1000). Finally sections were washed with 1x PBS, rinsed with water and mounted with Dako Fluorescent Mounting Medium (Dako). Mounted slides were analyzed with Nikon Eclipse TE2000-U microscope. Pictures were captured with Q Imaging Fast 1394 digital camera using QCapture Pro software (Version 6.0.0.412). Merges were digitally processed using Adobe Photoshop 11.0.

### Phenotypic characterization

In order to minimize the error due to sex or age differences all the phenotypic characterization was performed in adult male mice, of 5–9 months of age.

Albuminuria was measured in fresh urine samples. Albuminuria normalized by creatininuria (µg/mg) was determined as described previously [43]. The number of mice analyzed for each genotype was as follows: non transgenic mice: n = 10, transgenic 419 n = 9, 421 n = 9, 615 n = 3, 616 n = 13, 73a n = 17, 75a n = 9.

For histopathological analysis mice were transcardially perfused with 15 ml room temperature 1x PBS and then 30 ml of cold 4% PFA. Perfused kidney were dissected, dehydrated in alcohol gradient and embedded in paraffin. Four µm sections were used for periodic acid-Schiff reagent (PAS) staining. The number of mice analyzed for each genotype was as follows: non transgenic mice: n = 9, transgenic 419 n = 10, 421 n = 7, 615 n = 11, 616 n = 12, 73a n = 11, 75a n = 7. All of the samples were examined by two independent pathologists (Carpio and Ruiz) without knowledge of the genotypes. The pathological abnormalities in the kidney were graded based on the presence and severity of component abnormalities, including glomerulosclerosis, mesangial expansion, chronic inflammation, tubular atrophy or casts, fibrosis, and vascular injury. Grading for each component was performed using a semiquantitative scale as previously described [44] where zero was no abnormality and where one, two, three, and four represented mild, moderate, moderately severe, and severe abnormalities, respectively. The percentage of glomerular abnormalities is defined as the number of glomeruli with damage divided by the total number of glomeruli in the section.

For electron microscopy, kidney cortex samples were fixed in glutaraldehyde 2%. Specimens were rinsed in phosphate buffer, followed by OsO_4_, dehydrated in ethanol and acetone and embedded in Epon resin. Semi-thin sections were dyed with blue toluidine to evaluate general renal morphology. Afterwards, ultrathin sections were cut in a Sorvall microtome, incubated in uranyl acetate and a plumb solution and then visualized with a Hitachi H700 microscope. Sections were analyzed in blind by a specialized patho-nephrologist.

### Statistical analysis

T-test was performed to determine the statistical significance of the different parameters presented in this study between each transgenic line and the control group. Error bars represent standard errors of the mean. Data are expressed as mean ± SEM. Values of *P*<0.05 were considered to be significant.
